# Bedaquiline and Linezolid for Extensively Drug-Resistant Tuberculosis in Pregnant Woman

**DOI:** 10.3201/eid2310.161398

**Published:** 2017-10

**Authors:** Marie Jaspard, Elisabeth Elefant-Amoura, Isabelle Melonio, Inés De Montgolfier, Nicolas Veziris, Eric Caumes

**Affiliations:** Hôpital Pitié Salpêtrière, Paris, France (M. Jaspard, I. Melonio, N. Veziris, E. Caumes);; Hôpital Trousseau, Paris (I. De Montgolfier, E. Elefant-Amoura);; \Pierre and Marie Curie University of Sorbonne Université, Paris (N. Veziris)

**Keywords:** MDR TB, XDR TB, pregnancy, linezolid, bedaquiline, *Mycobacterium tuberculosis*, multidrug-resistant tuberculosis, tuberculosis, bacteria, mycobacteria, Georgia, France, antimicrobial resistance, respiratory infections, case study

## Abstract

A woman with extremely drug-resistant tuberculosis treated with a drug regimen including linezolid and bedaquiline during her last 3 weeks of pregnancy gave birth to a child without abnormalities. No fetal toxicities were noted by 2 years after delivery. This drug combination might be safe during the late third trimester of pregnancy.

In 2014, a total of 480,000 cases of multidrug-resistant tuberculosis (MDR TB) were present worldwide; 9% of these cases were also resistant to fluoroquinolones and aminoglycosides (1 of the 3 available injectable second-line drugs), thereby defining them as extensively drug-resistant TB (XDR TB) (*1*). Pregnant women with untreated TB have a mortality rate of 40%, suggesting a severe disease course in these women (*2*). Considering the severity of XDR TB, pregnant women with this form of TB need to be effectively treated before delivery. However, treatment regimens are usually stopped for fear of fetal toxicity (*3*). 

Bedaquiline efficacy has been demonstrated in MDR TB and is now recommended by the World Health Organization for the treatment of MDR TB (*4*). However, the half-life of bedaquiline in plasma is extremely prolonged, and serious side effects such as hepatitis and QT-interval prolongation can occur (*5*). Similarly, linezolid is an effective drug for treating XDR TB, but side effects such as neurotoxicity and hematologic toxicity frequently occur (*6*). Because no data are available on the treatment of pregnant woman with these drugs, bedaquiline and linezolid are not prescribed for this population. 

We report the case of a pregnant woman with XDR TB treated with a regimen including bedaquiline and linezolid. In 2008, MDR TB was diagnosed in a 33-year-old woman in Georgia. She was given a 7-drug regimen including ethambutol, pyrazinamide, cycloserine, para-aminosalicylic acid (PAS), and kanamycin for 18 months. In 2012, she relapsed and was given a different regimen of drugs (pyrazinamide, cycloserine, PAS, amoxicillin/clavulanate, capreomycin, levofloxacin, and prothionamide) for the same duration. In 2014, she relapsed again but with an XDR strain and was prescribed pyrazinamide, cycloserine, PAS, amoxicillin/clavulanate, capreomycin, levofloxacin, prothionamide, clarithromycin, and clofazimine. Despite 4 months of this drug regimen, sputum test results were positive. She eventually went to France for treatment.

When she arrived at Hôpital Pitié Salpêtrière she was 31 weeks pregnant. She had not received any MDR TB drugs since the beginning of her pregnancy. Her clinical status was good, except for a chronic cough over the past 6 months without weight loss. The fetus had no abnormalities. The woman’s sputum had >100 acid-fast bacilli and was culture positive for a *Mycobacterium tuberculosis* strain exhibiting resistance to isoniazid, rifampin, fluoroquinolones (low-level resistance), ethambutol, ethionamide, and aminoglycoside but susceptibility to cycloserine, PAS, bedaquiline, and linezolid. A computed tomography scan showed a large cavity in her upper left lung.

On the basis of the patient’s resistance profile, she was given the following drug regimen at 36 weeks’ gestation: bedaquiline, linezolid (600 mg/d), PAS, cycloserine, and levofloxacin. The Reference Centre for Teratogenic Agents (Paris, France) and the manufacturer of bedaquiline approved giving this regimen because the fetus did not have major side effects. 

Fetal status was monitored weekly. Delivery was planned at 39 weeks, and the patient gave birth to a healthy girl who was immediately separated from her mother because of the acid-fast bacilli in her mother’s sputum. 

Mycobacterial analysis of the placenta was negative. The newborn underwent 3 gastric washings to collect biological samples for further TB testing. These samples were negative by acid-fast staining and mycobacteria culture. Tuberculin skin test results were negative, and radiographs of her chest and cardiac ultrasonography gave unremarkable results. She did not have a QT prolongation or hepatitis.

After 24 months of therapy and lung surgery, the mother’s TB resolved. At 2 years of age, the child showed normal growth and did not have TB or signs of any clinical disorders, especially those of neurologic and cardiac conditions.

This report suggests that pregnant women with XDR TB can receive bedaquiline and linezolid during the last 3 weeks (late third trimester) of pregnancy without major side effects. Second-line therapy appears to be effective and safe during pregnancy and could be considered to treat some pregnant women with MDR TB. Indeed, in a study in Peru, no difference in efficacy was found when comparing nonpregnant women and pregnant women with MDR TB treated with second-line TB drugs (*7*). Also, long-term follow-up of children born to women given these treatments affirms the safety of second-line TB drugs during pregnancy (*8*,*9*).

However, treating pregnant women with XDR TB is more challenging. Our patient was given a regimen that included bedaquiline and linezolid, neither of which has its data available on safety during pregnancy. Even though the newborn was in good health at birth, no general conclusion could be drawn about the potential teratogenicity of these drugs because the treatment had been introduced only 3 weeks before delivery. In this single case, no specific maternal or fetal side effects were noticed, indicating the potential for using this drug combination. However, more data are needed to ensure the safety of these drugs during pregnancy. 
